# 2-Amino­pyridinium trifluoro­acetate

**DOI:** 10.1107/S1600536810006392

**Published:** 2010-02-27

**Authors:** Madhukar Hemamalini, Hoong-Kun Fun

**Affiliations:** a X-ray Crystallography Unit, School of Physics, Universiti Sains Malaysia, 11800 USM, Penang, Malaysia

## Abstract

The asymmetric unit of the title compound, C_5_H_7_N_2_^+^·C_2_F_3_O_2_^−^, contains four independent 2-amino­pyridinium cations and four independent trifluoro­acetate anions. In the crystal structure, these ions are linked by N—H⋯O hydrogen bonds, forming four cation–anion pairs each containing an *R*_2_^2^(8) ring motif. The ion pairs are linked into two independent chains along [100] by N—H⋯O hydrogen bonds. In addition, C—H⋯O and C—H⋯F hydrogen bonds and π⋯π inter­actions [centoid–centroid separation = 3.6007 (17) Å] are observed.

## Related literature

For background to the chemistry of substituted pyridines, see: Pozharski *et al.* (1997 ▶); Katritzky *et al.* (1996 ▶). For related structures, see: Chao *et al.* (1975 ▶); Gellert & Hsu (1988 ▶); Demir *et al.* (2005 ▶); Jebas *et al.* (2006 ▶); Rademeyer (2007 ▶); Windholz (1976 ▶). For details of hydrogen bonding, see: Jeffrey & Saenger (1991 ▶); Jeffrey (1997 ▶); Scheiner (1997 ▶). For hydrogen-bond motifs, see: Bernstein *et al.* (1995 ▶). For the stability of the temperature controller used in the data collection, see: Cosier & Glazer (1986 ▶).
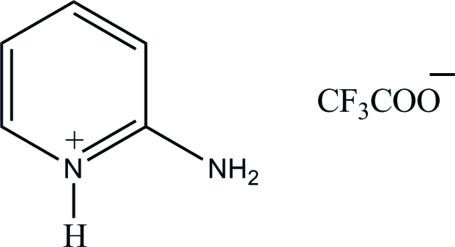


## Experimental

### 

#### Crystal data

C_5_H_7_N_2_^+^·C_2_F_3_O_2_^−^
*M* *_r_* = 208.15
Monoclinic, 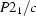

*a* = 11.4641 (15) Å
*b* = 10.0221 (13) Å
*c* = 29.928 (4) Å
β = 92.918 (3)°
*V* = 3434.1 (8) Å^3^
*Z* = 16
Mo *K*α radiation
μ = 0.16 mm^−1^
*T* = 100 K
0.35 × 0.17 × 0.04 mm

#### Data collection

Bruker APEX DUO CCD area-detector diffractometer
Absorption correction: multi-scan (*SADABS*; Bruker, 2009 ▶) *T*_min_ = 0.947, *T*_max_ = 0.994
28669 measured reflections
6732 independent reflections
4154 reflections with *I* > 2σ(*I*)
*R*_int_ = 0.072

#### Refinement

*R*[*F* ^2^ > 2σ(*F* ^2^)] = 0.044
*wR*(*F* ^2^) = 0.141
*S* = 1.02
6732 reflections
617 parameters
All H-atom parameters refined
Δρ_max_ = 0.44 e Å^−3^
Δρ_min_ = −0.44 e Å^−3^

### 

Data collection: *APEX2* (Bruker, 2009 ▶); cell refinement: *SAINT* (Bruker, 2009 ▶); data reduction: *SAINT*; program(s) used to solve structure: *SHELXTL* (Sheldrick, 2008 ▶); program(s) used to refine structure: *SHELXTL*; molecular graphics: *SHELXTL*; software used to prepare material for publication: *SHELXTL* and *PLATON* (Spek, 2009 ▶).

## Supplementary Material

Crystal structure: contains datablocks global, I. DOI: 10.1107/S1600536810006392/ci5036sup1.cif

Structure factors: contains datablocks I. DOI: 10.1107/S1600536810006392/ci5036Isup2.hkl

Additional supplementary materials:  crystallographic information; 3D view; checkCIF report

## Figures and Tables

**Table 1:** Hydrogen-bond geometry (Å, °)

*D*—H⋯*A*	*D*—H	H⋯*A*	*D*⋯*A*	*D*—H⋯*A*
N1*A*—H1*NA*⋯O2*D*^i^	0.92 (3)	1.91 (3)	2.809 (3)	168 (2)
N2*A*—H2*NA*⋯O1*D*^i^	1.02 (4)	1.79 (4)	2.795 (4)	169 (4)
N2*A*—H3*NA*⋯O1*A*^ii^	0.84 (3)	2.10 (3)	2.899 (3)	160 (3)
N1*B*—H1*NB*⋯O2*C*^iii^	0.97 (3)	1.75 (3)	2.704 (3)	166 (3)
N2*B*—H2*NB*⋯O1*C*^iii^	0.91 (4)	2.01 (4)	2.892 (4)	164 (3)
N2*B*—H3*NB*⋯O2*B*^iii^	0.86 (3)	2.07 (3)	2.858 (3)	154 (3)
N1*C*—H1*NC*⋯O1*A*^iv^	0.86 (3)	1.94 (3)	2.789 (3)	172 (3)
N2*C*—H2*NC*⋯O2*A*^iv^	0.90 (3)	1.93 (3)	2.827 (4)	177 (3)
N2*C*—H3*NC*⋯O2*D*^v^	0.94 (3)	2.04 (3)	2.894 (3)	150 (3)
N1*D*—H1*ND*⋯O2*B*^vi^	0.92 (3)	1.80 (3)	2.701 (3)	164 (2)
N2*D*—H2*ND*⋯O1*B*^vi^	0.95 (3)	1.97 (3)	2.878 (4)	160 (2)
N2*D*—H3*ND*⋯O2*C*^vii^	0.84 (3)	2.21 (3)	2.908 (3)	141 (3)
C1*A*—H1*A*⋯O2*A*	1.00 (3)	2.20 (3)	3.141 (3)	155 (2)
C1*B*—H1*B*⋯F3*C*^viii^	1.06 (3)	2.43 (3)	3.288 (4)	137 (2)
C1*C*—H1*C*⋯O1*D*^ix^	0.95 (2)	2.21 (2)	3.107 (3)	158 (2)
C4*A*—H4*A*⋯F1*A*^ii^	1.06 (3)	2.34 (3)	3.352 (3)	159 (2)
C4*B*—H4*B*⋯O1*C*	1.05 (3)	2.25 (3)	3.294 (4)	171 (2)
C4*C*—H4*C*⋯F3*D*^v^	0.93 (3)	2.50 (3)	3.365 (3)	155 (3)
C4*D*—H4*D*⋯O1*B*^ii^	1.08 (2)	2.18 (2)	3.204 (4)	159 (2)

Symmetry codes: (i) 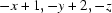; (ii) 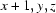; (iii) 
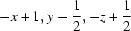
; (iv) 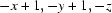; (v) 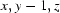; (vi) 
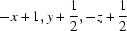
; (vii) 
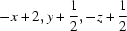
; (viii) 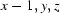; (ix) 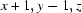.

**Fig. 1.**
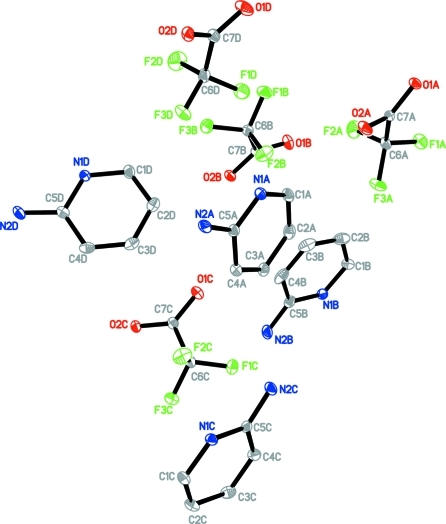
The asymmetric unit of the title compound. Displacement ellipsoids are drawn at the 30% probability level. H atoms have been omitted for clarity.

**Fig. 2.**
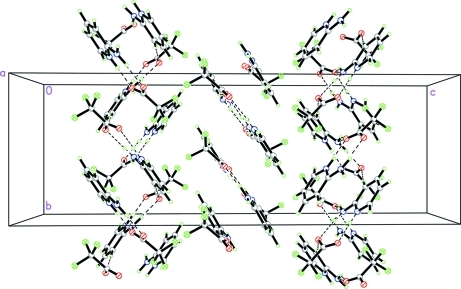
The crystal packing of the title compound, showing hydrogen-bonded (dashed lines) networks.

## supplementary crystallographic information

### Comment

Pyridine and its derivatives play an important role in heterocyclic chemistry (Pozharski *et al.*, 1997; Katritzky *et al.*, 1996). They are often involved in hydrogen-bond interactions (Jeffrey & Saenger, 1991; Jeffrey, 1997; Scheiner, 1997). 2-Aminopyridine is used in the manufacture of pharmaceuticals, especially antihistaminic drugs (Windholz, 1976). The crystal structures of 2-aminopyridine (Chao *et al.*, 1975), 2-aminopyridinium salicylate (Gellert & Hsu, 1988), 2-amino-pyridinium dihydrogenphosphate (Demir *et al.*, 2005), bis(2-aminopyridinium) sulfate (Jebas *et al.*, 2006) and 2-aminopyridinium nitrate (Rademeyer, 2007) have been reported in the literature. The crystal structure determination of the title compound was undertaken to study the hydrogen bonding interactions in it.

The asymmetric unit of the title compound consists of four crystallographically independent 2-aminopyridinium cations (A, B, C & D) and four trifluoroacetate anions (A, B, C & D) (Fig. 1).  Each 2-aminopyridinium cation is planar, with a maximum deviation of 0.017 (3) Å for atom N2A (molecule A), 0.007 (2) Å for atom C3B (molecule B), 0.007 (2) Å  for atom N1C (molecule C) and 0.008 (3) Å for atom C5D (molecule D).

In the crystal structure (Fig. 2), carboxylate groups of A, B, C and D trifluoroacetate anions interact with two N–H groups of D, C, A and B 2-aminopyridinium cations, respectively, via pairs of N—H···O hydrogen bonds generating *R*_2_^2^(8) motifs (Bernstein *et al.*, 1995). The ionic pairs are linked into chains along [100] by N—H···O hydrogen bonds involving the remaining N–H groups. The crystal structure is further stabilized by C—H···O and C—H···F hydrogen bonds (Table 1) and π···π interactions involving the N1A/C1A–C5A pyridine rings at (x, y, z) and (1-x, 1-y, -z) with a centoid-to-centroid separation of 3.6007 (17) Å.

### Experimental

To a hot methanol solution (20 ml) of 2-aminopyridine (47 mg, Aldrich) was added a few drops of trifluoroacetic acid.  The solution was warmed over a water bath for a few minutes. The resulting solution was allowed to cool slowly to room temperature. Crystals of the title compound appeared from the mother liquor after a few days.

### Refinement

All the H atoms were located in a difference Fourier map and allowed to refine freely [N–H = 0.83 (4)–1.02 (4) Å, C–H = 0.91 (3)–1.08 (3) Å].

### Crystal data

**Table:**

C_5_H_7_N_2_^+^·C_2_F_3_O_2_^−^	*F*(000) = 1696
*M**_r_* = 208.15	*D*_x_ = 1.610 Mg m^−^^3^
Monoclinic, *P*2_1_/*c*	Mo *K*α radiation, λ = 0.71073 Å
Hall symbol: -P 2ybc	Cell parameters from 3066 reflections
*a* = 11.4641 (15) Å	θ = 2.7–24.0°
*b* = 10.0221 (13) Å	µ = 0.16 mm^−^^1^
*c* = 29.928 (4) Å	*T* = 100 K
β = 92.918 (3)°	Plate, colourless
*V* = 3434.1 (8) Å^3^	0.35 × 0.17 × 0.04 mm
*Z* = 16	

### Data collection

**Table:**

Bruker APEX DUO CCD area-detector diffractometer	6732 independent reflections
Radiation source: fine-focus sealed tube	4154 reflections with *I* > 2σ(*I*)
graphite	*R*_int_ = 0.072
φ and ω scans	θ_max_ = 26.0°, θ_min_ = 1.4°
Absorption correction: multi-scan (*SADABS*; Bruker, 2009)	*h* = −14→12
*T*_min_ = 0.947, *T*_max_ = 0.994	*k* = −12→12
28669 measured reflections	*l* = −36→36

### Refinement

**Table:**

Refinement on *F*^2^	Primary atom site location: structure-invariant direct methods
Least-squares matrix: full	Secondary atom site location: difference Fourier map
*R*[*F*^2^ > 2σ(*F*^2^)] = 0.044	Hydrogen site location: inferred from neighbouring sites
*wR*(*F*^2^) = 0.141	All H-atom parameters refined
*S* = 1.02	*w* = 1/[σ^2^(*F*_o_^2^) + (0.072*P*)^2^] where *P* = (*F*_o_^2^ + 2*F*_c_^2^)/3
6732 reflections	(Δ/σ)_max_ = 0.001
617 parameters	Δρ_max_ = 0.44 e Å^−^^3^
0 restraints	Δρ_min_ = −0.44 e Å^−^^3^

### Special details

**Table:**

Experimental. The crystal was placed in the cold stream of an Oxford Cryosystems Cobra open-flow nitrogen cryostat (Cosier & Glazer, 1986) operating at 100.0 (1) K.
Geometry. All s.u.'s (except the s.u. in the dihedral angle between two l.s. planes) are estimated using the full covariance matrix. The cell s.u.'s are taken into account individually in the estimation of s.u.'s in distances, angles and torsion angles; correlations between s.u.'s in cell parameters are only used when they are defined by crystal symmetry. An approximate (isotropic) treatment of cell s.u.'s is used for estimating s.u.'s involving l.s. planes.
Refinement. Refinement of F^2^ against ALL reflections. The weighted R-factor wR and goodness of fit S are based on F^2^, conventional R-factors R are based on F, with F set to zero for negative F^2^. The threshold expression of F^2^ > 2σ(F^2^) is used only for calculating R-factors(gt) etc. and is not relevant to the choice of reflections for refinement. R-factors based on F^2^ are statistically about twice as large as those based on F, and R- factors based on ALL data will be even larger.

### Fractional atomic coordinates and isotropic or equivalent isotropic displacement parameters (Å^2^)

**Table:**

	*x*	*y*	*z*	*U*_iso_*/*U*_eq_	
N1A	0.53238 (18)	0.6375 (2)	0.03514 (8)	0.0211 (5)	
N2A	0.7286 (2)	0.6627 (3)	0.02401 (9)	0.0266 (6)	
C1A	0.4393 (2)	0.5710 (3)	0.05182 (10)	0.0248 (7)	
C2A	0.4546 (3)	0.4564 (3)	0.07439 (10)	0.0271 (7)	
C3A	0.5680 (2)	0.4031 (3)	0.08038 (10)	0.0246 (7)	
C4A	0.6604 (2)	0.4684 (3)	0.06365 (10)	0.0248 (7)	
C5A	0.6430 (2)	0.5902 (3)	0.04029 (9)	0.0218 (6)	
F1A	−0.04773 (14)	0.44273 (16)	0.07172 (5)	0.0314 (4)	
F2A	0.06503 (16)	0.56750 (19)	0.11269 (6)	0.0419 (5)	
F3A	0.13664 (14)	0.40701 (17)	0.07542 (6)	0.0347 (4)	
O1A	−0.01983 (15)	0.65268 (18)	0.01836 (6)	0.0218 (4)	
O2A	0.17434 (15)	0.6235 (2)	0.02589 (7)	0.0289 (5)	
C6A	0.0564 (2)	0.5042 (3)	0.07342 (9)	0.0231 (6)	
C7A	0.0723 (2)	0.6020 (3)	0.03494 (9)	0.0187 (6)	
N1B	0.25015 (19)	0.0690 (2)	0.22063 (8)	0.0223 (5)	
N2B	0.4426 (2)	0.0544 (3)	0.24561 (10)	0.0327 (7)	
C1B	0.1593 (3)	0.1252 (3)	0.19635 (10)	0.0290 (7)	
C2B	0.1754 (3)	0.2363 (3)	0.17180 (11)	0.0352 (8)	
C3B	0.2877 (3)	0.2902 (3)	0.17187 (11)	0.0365 (8)	
C4B	0.3786 (3)	0.2344 (3)	0.19497 (11)	0.0342 (8)	
C5B	0.3600 (2)	0.1179 (3)	0.22101 (10)	0.0265 (7)	
F1B	0.19140 (15)	0.79602 (17)	0.16527 (6)	0.0357 (4)	
F2B	0.31202 (16)	0.64578 (17)	0.14540 (6)	0.0391 (5)	
F3B	0.36394 (14)	0.78893 (16)	0.19615 (6)	0.0326 (4)	
O1B	0.12827 (16)	0.5810 (2)	0.21178 (7)	0.0306 (5)	
O2B	0.30890 (15)	0.57592 (19)	0.24392 (6)	0.0237 (5)	
C6B	0.2742 (2)	0.7125 (3)	0.18099 (10)	0.0240 (7)	
C7B	0.2322 (2)	0.6146 (3)	0.21597 (9)	0.0220 (6)	
N1C	0.97689 (18)	0.1324 (2)	0.03769 (8)	0.0195 (5)	
N2C	0.7775 (2)	0.1534 (3)	0.02793 (9)	0.0234 (6)	
C1C	1.0764 (2)	0.0718 (3)	0.05421 (10)	0.0233 (7)	
C2C	1.0705 (3)	−0.0409 (3)	0.07859 (10)	0.0259 (7)	
C3C	0.9596 (3)	−0.0928 (3)	0.08655 (10)	0.0259 (7)	
C4C	0.8602 (2)	−0.0311 (3)	0.07060 (10)	0.0229 (6)	
C5C	0.8686 (2)	0.0864 (3)	0.04501 (9)	0.0187 (6)	
F1C	0.70647 (15)	0.14098 (17)	0.14889 (6)	0.0361 (5)	
F2C	0.83699 (16)	0.28461 (17)	0.13276 (6)	0.0383 (5)	
F3C	0.87288 (13)	0.14044 (16)	0.18453 (5)	0.0290 (4)	
O1C	0.64277 (16)	0.3622 (2)	0.19178 (7)	0.0296 (5)	
O2C	0.81484 (15)	0.35674 (19)	0.23072 (6)	0.0243 (5)	
C6C	0.7897 (2)	0.2205 (3)	0.16693 (9)	0.0235 (7)	
C7C	0.7438 (2)	0.3217 (3)	0.20015 (9)	0.0215 (6)	
N1D	0.75292 (18)	0.8656 (2)	0.20606 (8)	0.0203 (5)	
N2D	0.9457 (2)	0.8664 (3)	0.23238 (9)	0.0272 (6)	
C1D	0.6606 (2)	0.8152 (3)	0.18099 (10)	0.0252 (7)	
C2D	0.6725 (3)	0.7041 (3)	0.15644 (10)	0.0293 (7)	
C3D	0.7819 (3)	0.6428 (3)	0.15695 (10)	0.0283 (7)	
C4D	0.8744 (3)	0.6922 (3)	0.18151 (10)	0.0253 (7)	
C5D	0.8595 (2)	0.8081 (3)	0.20725 (9)	0.0216 (6)	
F1D	0.38550 (14)	0.89359 (17)	0.07557 (6)	0.0363 (5)	
F2D	0.45932 (18)	1.0533 (2)	0.11547 (6)	0.0484 (5)	
F3D	0.56876 (14)	0.93681 (17)	0.07456 (6)	0.0355 (5)	
O1D	0.33350 (16)	1.1182 (2)	0.02989 (8)	0.0394 (6)	
O2D	0.52497 (15)	1.14436 (19)	0.02069 (6)	0.0238 (5)	
C6D	0.4630 (2)	0.9937 (3)	0.07548 (10)	0.0261 (7)	
C7D	0.4378 (2)	1.0945 (3)	0.03804 (10)	0.0245 (7)	
H1A	0.364 (3)	0.615 (3)	0.0418 (9)	0.026 (8)*	
H2A	0.383 (3)	0.406 (3)	0.0863 (11)	0.050 (10)*	
H3A	0.579 (3)	0.315 (3)	0.0972 (10)	0.037 (9)*	
H4A	0.748 (3)	0.435 (3)	0.0678 (9)	0.031 (8)*	
H1NA	0.525 (2)	0.710 (3)	0.0166 (9)	0.016 (7)*	
H2NA	0.697 (4)	0.743 (4)	0.0063 (13)	0.080 (14)*	
H3NA	0.799 (3)	0.641 (3)	0.0250 (11)	0.052 (11)*	
H1B	0.076 (3)	0.081 (3)	0.2000 (10)	0.042 (9)*	
H2B	0.100 (3)	0.280 (3)	0.1548 (11)	0.057 (11)*	
H3B	0.297 (3)	0.364 (3)	0.1548 (11)	0.041 (10)*	
H4B	0.466 (3)	0.266 (3)	0.1957 (10)	0.044 (9)*	
H1NB	0.240 (3)	−0.011 (3)	0.2385 (9)	0.031 (9)*	
H2NB	0.430 (3)	−0.007 (4)	0.2673 (11)	0.043 (11)*	
H3NB	0.511 (3)	0.087 (3)	0.2509 (10)	0.038 (10)*	
H1C	1.148 (2)	0.110 (3)	0.0459 (9)	0.022 (8)*	
H2C	1.139 (3)	−0.086 (3)	0.0906 (10)	0.035 (9)*	
H3C	0.955 (2)	−0.174 (3)	0.1037 (10)	0.029 (8)*	
H4C	0.787 (3)	−0.064 (3)	0.0765 (9)	0.027 (8)*	
H1NC	0.984 (3)	0.202 (3)	0.0213 (10)	0.029 (9)*	
H2NC	0.792 (3)	0.222 (3)	0.0099 (11)	0.049 (11)*	
H3NC	0.701 (3)	0.121 (4)	0.0304 (12)	0.062 (12)*	
H1D	0.583 (2)	0.864 (3)	0.1846 (9)	0.023 (7)*	
H2D	0.603 (3)	0.670 (3)	0.1391 (10)	0.034 (8)*	
H3D	0.791 (2)	0.560 (3)	0.1406 (9)	0.027 (8)*	
H4D	0.961 (2)	0.650 (3)	0.1830 (9)	0.027 (8)*	
H1ND	0.746 (2)	0.938 (3)	0.2247 (9)	0.026 (8)*	
H2ND	0.940 (2)	0.937 (3)	0.2533 (10)	0.026 (8)*	
H3ND	1.009 (3)	0.826 (3)	0.2377 (10)	0.032 (9)*	

### Atomic displacement parameters (Å^2^)

**Table:**

	*U*^11^	*U*^22^	*U*^33^	*U*^12^	*U*^13^	*U*^23^
N1A	0.0145 (12)	0.0229 (14)	0.0259 (14)	0.0006 (10)	0.0017 (10)	−0.0020 (12)
N2A	0.0104 (12)	0.0265 (15)	0.0431 (17)	0.0006 (10)	0.0041 (11)	−0.0005 (12)
C1A	0.0138 (14)	0.0332 (18)	0.0275 (17)	−0.0003 (12)	0.0023 (12)	−0.0038 (14)
C2A	0.0248 (16)	0.0342 (18)	0.0227 (17)	−0.0062 (13)	0.0035 (12)	−0.0053 (14)
C3A	0.0236 (15)	0.0272 (17)	0.0230 (17)	−0.0034 (13)	0.0014 (12)	−0.0061 (14)
C4A	0.0209 (15)	0.0246 (16)	0.0285 (17)	0.0018 (12)	−0.0021 (12)	−0.0022 (14)
C5A	0.0171 (14)	0.0240 (16)	0.0246 (16)	−0.0012 (11)	0.0020 (11)	−0.0063 (13)
F1A	0.0229 (9)	0.0390 (11)	0.0329 (10)	−0.0065 (7)	0.0054 (7)	0.0108 (8)
F2A	0.0512 (12)	0.0532 (13)	0.0209 (10)	−0.0013 (9)	−0.0012 (8)	−0.0068 (9)
F3A	0.0275 (9)	0.0362 (10)	0.0404 (11)	0.0104 (8)	0.0016 (8)	0.0155 (9)
O1A	0.0134 (9)	0.0236 (11)	0.0283 (11)	0.0014 (8)	0.0007 (8)	0.0043 (9)
O2A	0.0123 (10)	0.0337 (12)	0.0409 (13)	−0.0013 (8)	0.0015 (8)	0.0128 (10)
C6A	0.0167 (14)	0.0278 (17)	0.0247 (17)	−0.0001 (12)	−0.0005 (11)	0.0022 (14)
C7A	0.0153 (14)	0.0185 (15)	0.0222 (15)	−0.0001 (11)	−0.0010 (11)	−0.0006 (12)
N1B	0.0181 (12)	0.0207 (13)	0.0285 (14)	−0.0021 (10)	0.0046 (10)	0.0000 (11)
N2B	0.0184 (14)	0.0388 (17)	0.0407 (18)	−0.0071 (12)	0.0006 (12)	−0.0067 (15)
C1B	0.0272 (16)	0.0269 (17)	0.0329 (19)	0.0056 (13)	0.0017 (13)	−0.0004 (14)
C2B	0.046 (2)	0.0289 (19)	0.0311 (19)	0.0078 (15)	0.0098 (15)	0.0050 (15)
C3B	0.056 (2)	0.0247 (19)	0.031 (2)	0.0006 (16)	0.0200 (16)	0.0015 (16)
C4B	0.043 (2)	0.0269 (18)	0.0346 (19)	−0.0140 (15)	0.0184 (15)	−0.0088 (15)
C5B	0.0237 (16)	0.0253 (17)	0.0312 (18)	−0.0036 (13)	0.0088 (13)	−0.0102 (14)
F1B	0.0408 (11)	0.0322 (10)	0.0334 (11)	0.0069 (8)	−0.0045 (8)	0.0080 (8)
F2B	0.0535 (12)	0.0365 (11)	0.0288 (11)	0.0006 (9)	0.0179 (8)	−0.0056 (9)
F3B	0.0310 (10)	0.0300 (10)	0.0369 (11)	−0.0055 (8)	0.0029 (8)	0.0029 (8)
O1B	0.0203 (11)	0.0425 (13)	0.0286 (12)	−0.0044 (9)	−0.0027 (8)	0.0026 (10)
O2B	0.0171 (10)	0.0271 (11)	0.0267 (11)	0.0021 (8)	−0.0008 (8)	0.0026 (9)
C6B	0.0215 (15)	0.0244 (16)	0.0258 (17)	0.0021 (12)	−0.0011 (12)	−0.0041 (13)
C7B	0.0207 (15)	0.0248 (16)	0.0205 (16)	0.0028 (12)	0.0023 (12)	−0.0052 (13)
N1C	0.0163 (12)	0.0183 (13)	0.0239 (14)	0.0000 (9)	0.0024 (10)	0.0029 (11)
N2C	0.0123 (12)	0.0249 (15)	0.0330 (16)	−0.0029 (10)	0.0013 (10)	0.0035 (12)
C1C	0.0156 (15)	0.0299 (17)	0.0245 (16)	0.0007 (12)	0.0013 (11)	0.0016 (13)
C2C	0.0219 (15)	0.0303 (18)	0.0253 (17)	0.0084 (13)	0.0003 (12)	0.0050 (14)
C3C	0.0346 (17)	0.0183 (16)	0.0254 (17)	0.0011 (13)	0.0074 (13)	0.0061 (13)
C4C	0.0198 (15)	0.0208 (16)	0.0287 (17)	−0.0012 (12)	0.0071 (12)	0.0006 (13)
C5C	0.0159 (14)	0.0187 (15)	0.0216 (15)	−0.0028 (11)	0.0025 (11)	−0.0027 (12)
F1C	0.0356 (10)	0.0326 (10)	0.0391 (11)	−0.0034 (8)	−0.0100 (8)	−0.0083 (9)
F2C	0.0545 (12)	0.0334 (11)	0.0283 (11)	−0.0007 (9)	0.0146 (8)	0.0082 (8)
F3C	0.0258 (9)	0.0295 (10)	0.0316 (10)	0.0060 (7)	0.0007 (7)	0.0014 (8)
O1C	0.0192 (11)	0.0336 (12)	0.0354 (13)	0.0032 (9)	−0.0037 (9)	0.0000 (10)
O2C	0.0172 (10)	0.0266 (11)	0.0288 (12)	−0.0003 (8)	−0.0025 (8)	−0.0050 (9)
C6C	0.0214 (15)	0.0242 (16)	0.0247 (16)	−0.0021 (12)	−0.0011 (12)	0.0063 (13)
C7C	0.0197 (15)	0.0220 (16)	0.0228 (16)	−0.0031 (11)	0.0017 (12)	0.0039 (12)
N1D	0.0162 (12)	0.0202 (13)	0.0244 (14)	0.0004 (10)	0.0007 (10)	−0.0004 (11)
N2D	0.0155 (13)	0.0337 (16)	0.0322 (16)	0.0024 (11)	−0.0021 (11)	0.0024 (13)
C1D	0.0200 (15)	0.0279 (17)	0.0277 (17)	−0.0038 (12)	0.0007 (12)	0.0021 (14)
C2D	0.0313 (17)	0.0258 (17)	0.0310 (18)	−0.0058 (13)	0.0026 (14)	−0.0024 (14)
C3D	0.0374 (18)	0.0200 (17)	0.0283 (18)	−0.0011 (14)	0.0080 (14)	−0.0003 (14)
C4D	0.0295 (17)	0.0217 (16)	0.0256 (17)	0.0053 (13)	0.0093 (13)	0.0057 (13)
C5D	0.0199 (15)	0.0238 (16)	0.0214 (16)	0.0005 (11)	0.0052 (11)	0.0088 (12)
F1D	0.0292 (10)	0.0351 (10)	0.0449 (12)	−0.0034 (8)	0.0047 (8)	0.0097 (9)
F2D	0.0641 (14)	0.0538 (13)	0.0277 (11)	0.0036 (10)	0.0064 (9)	−0.0105 (10)
F3D	0.0227 (9)	0.0446 (11)	0.0386 (11)	0.0077 (8)	−0.0039 (7)	0.0054 (9)
O1D	0.0132 (11)	0.0437 (14)	0.0617 (16)	0.0031 (9)	0.0068 (10)	0.0201 (12)
O2D	0.0146 (10)	0.0283 (11)	0.0287 (12)	−0.0023 (8)	0.0046 (8)	−0.0017 (9)
C6D	0.0208 (15)	0.0291 (17)	0.0287 (18)	−0.0018 (12)	0.0025 (12)	−0.0077 (14)
C7D	0.0149 (14)	0.0272 (17)	0.0315 (17)	0.0002 (12)	0.0036 (12)	−0.0035 (14)

### Geometric parameters (Å, °)

**Table:**

N1A—C5A	1.355 (3)	N1C—C5C	1.353 (3)
N1A—C1A	1.373 (4)	N1C—C1C	1.363 (3)
N1A—H1NA	0.92 (3)	N1C—H1NC	0.86 (3)
N2A—C5A	1.334 (4)	N2C—C5C	1.323 (3)
N2A—H2NA	1.02 (4)	N2C—H2NC	0.90 (3)
N2A—H3NA	0.83 (4)	N2C—H3NC	0.94 (4)
C1A—C2A	1.340 (4)	C1C—C2C	1.348 (4)
C1A—H1A	1.00 (3)	C1C—H1C	0.95 (3)
C2A—C3A	1.409 (4)	C2C—C3C	1.406 (4)
C2A—H2A	1.04 (3)	C2C—H2C	0.96 (3)
C3A—C4A	1.362 (4)	C3C—C4C	1.361 (4)
C3A—H3A	1.02 (3)	C3C—H3C	0.97 (3)
C4A—C5A	1.416 (4)	C4C—C5C	1.410 (4)
C4A—H4A	1.06 (3)	C4C—H4C	0.92 (3)
F1A—C6A	1.342 (3)	F1C—C6C	1.336 (3)
F2A—C6A	1.335 (3)	F2C—C6C	1.345 (3)
F3A—C6A	1.339 (3)	F3C—C6C	1.334 (3)
O1A—C7A	1.252 (3)	O1C—C7C	1.240 (3)
O2A—C7A	1.233 (3)	O2C—C7C	1.244 (3)
C6A—C7A	1.531 (4)	C6C—C7C	1.533 (4)
N1B—C5B	1.351 (3)	N1D—C5D	1.349 (3)
N1B—C1B	1.361 (4)	N1D—C1D	1.363 (3)
N1B—H1NB	0.97 (3)	N1D—H1ND	0.92 (3)
N2B—C5B	1.331 (4)	N2D—C5D	1.346 (4)
N2B—H2NB	0.91 (3)	N2D—H2ND	0.95 (3)
N2B—H3NB	0.86 (3)	N2D—H3ND	0.84 (3)
C1B—C2B	1.352 (4)	C1D—C2D	1.345 (4)
C1B—H1B	1.06 (3)	C1D—H1D	1.03 (3)
C2B—C3B	1.396 (5)	C2D—C3D	1.395 (4)
C2B—H2B	1.07 (3)	C2D—H2D	0.99 (3)
C3B—C4B	1.342 (5)	C3D—C4D	1.353 (4)
C3B—H3B	0.91 (3)	C3D—H3D	0.97 (3)
C4B—C5B	1.426 (4)	C4D—C5D	1.409 (4)
C4B—H4B	1.05 (3)	C4D—H4D	1.08 (3)
F1B—C6B	1.334 (3)	F1D—C6D	1.340 (3)
F2B—C6B	1.348 (3)	F2D—C6D	1.340 (3)
F3B—C6B	1.343 (3)	F3D—C6D	1.341 (3)
O1B—C7B	1.238 (3)	O1D—C7D	1.231 (3)
O2B—C7B	1.245 (3)	O2D—C7D	1.253 (3)
C6B—C7B	1.530 (4)	C6D—C7D	1.526 (4)
			
C5A—N1A—C1A	121.9 (3)	C5C—N1C—C1C	123.2 (3)
C5A—N1A—H1NA	113.5 (17)	C5C—N1C—H1NC	119 (2)
C1A—N1A—H1NA	124.1 (17)	C1C—N1C—H1NC	118 (2)
C5A—N2A—H2NA	112 (2)	C5C—N2C—H2NC	117 (2)
C5A—N2A—H3NA	125 (2)	C5C—N2C—H3NC	121 (2)
H2NA—N2A—H3NA	123 (3)	H2NC—N2C—H3NC	121 (3)
C2A—C1A—N1A	121.1 (3)	C2C—C1C—N1C	120.4 (3)
C2A—C1A—H1A	127.9 (16)	C2C—C1C—H1C	123.0 (16)
N1A—C1A—H1A	110.7 (16)	N1C—C1C—H1C	116.5 (16)
C1A—C2A—C3A	119.0 (3)	C1C—C2C—C3C	118.2 (3)
C1A—C2A—H2A	120.0 (18)	C1C—C2C—H2C	122.2 (18)
C3A—C2A—H2A	120.9 (18)	C3C—C2C—H2C	119.6 (18)
C4A—C3A—C2A	120.1 (3)	C4C—C3C—C2C	121.4 (3)
C4A—C3A—H3A	121.1 (17)	C4C—C3C—H3C	119.9 (17)
C2A—C3A—H3A	118.8 (17)	C2C—C3C—H3C	118.7 (17)
C3A—C4A—C5A	120.2 (3)	C3C—C4C—C5C	119.4 (3)
C3A—C4A—H4A	123.7 (16)	C3C—C4C—H4C	121.2 (18)
C5A—C4A—H4A	116.1 (16)	C5C—C4C—H4C	119.4 (18)
N2A—C5A—N1A	118.0 (3)	N2C—C5C—N1C	118.5 (3)
N2A—C5A—C4A	124.2 (3)	N2C—C5C—C4C	124.0 (3)
N1A—C5A—C4A	117.8 (3)	N1C—C5C—C4C	117.5 (2)
F2A—C6A—F3A	106.7 (2)	F3C—C6C—F1C	106.4 (2)
F2A—C6A—F1A	106.1 (2)	F3C—C6C—F2C	106.2 (2)
F3A—C6A—F1A	106.0 (2)	F1C—C6C—F2C	106.6 (2)
F2A—C6A—C7A	110.6 (2)	F3C—C6C—C7C	113.8 (2)
F3A—C6A—C7A	113.1 (2)	F1C—C6C—C7C	113.3 (2)
F1A—C6A—C7A	113.9 (2)	F2C—C6C—C7C	110.0 (2)
O2A—C7A—O1A	129.3 (3)	O1C—C7C—O2C	128.8 (3)
O2A—C7A—C6A	115.3 (2)	O1C—C7C—C6C	115.7 (2)
O1A—C7A—C6A	115.3 (2)	O2C—C7C—C6C	115.5 (2)
C5B—N1B—C1B	122.8 (3)	C5D—N1D—C1D	122.2 (3)
C5B—N1B—H1NB	115.4 (17)	C5D—N1D—H1ND	115.3 (18)
C1B—N1B—H1NB	121.8 (17)	C1D—N1D—H1ND	122.3 (18)
C5B—N2B—H2NB	126 (2)	C5D—N2D—H2ND	128.0 (17)
C5B—N2B—H3NB	122 (2)	C5D—N2D—H3ND	120 (2)
H2NB—N2B—H3NB	108 (3)	H2ND—N2D—H3ND	109 (3)
C2B—C1B—N1B	120.6 (3)	C2D—C1D—N1D	120.6 (3)
C2B—C1B—H1B	123.2 (16)	C2D—C1D—H1D	124.2 (15)
N1B—C1B—H1B	116.0 (16)	N1D—C1D—H1D	115.0 (15)
C1B—C2B—C3B	118.0 (3)	C1D—C2D—C3D	118.3 (3)
C1B—C2B—H2B	117.9 (19)	C1D—C2D—H2D	118.3 (18)
C3B—C2B—H2B	124.0 (19)	C3D—C2D—H2D	123.4 (18)
C4B—C3B—C2B	122.0 (3)	C4D—C3D—C2D	121.6 (3)
C4B—C3B—H3B	121 (2)	C4D—C3D—H3D	118.7 (17)
C2B—C3B—H3B	117 (2)	C2D—C3D—H3D	119.6 (17)
C3B—C4B—C5B	119.3 (3)	C3D—C4D—C5D	119.0 (3)
C3B—C4B—H4B	126.8 (17)	C3D—C4D—H4D	124.8 (14)
C5B—C4B—H4B	113.9 (17)	C5D—C4D—H4D	116.2 (14)
N2B—C5B—N1B	117.9 (3)	N2D—C5D—N1D	117.9 (3)
N2B—C5B—C4B	124.9 (3)	N2D—C5D—C4D	123.9 (3)
N1B—C5B—C4B	117.3 (3)	N1D—C5D—C4D	118.2 (3)
F1B—C6B—F3B	106.3 (2)	F1D—C6D—F2D	106.3 (2)
F1B—C6B—F2B	106.5 (2)	F1D—C6D—F3D	106.4 (2)
F3B—C6B—F2B	106.2 (2)	F2D—C6D—F3D	106.1 (2)
F1B—C6B—C7B	113.6 (2)	F1D—C6D—C7D	113.4 (2)
F3B—C6B—C7B	113.4 (2)	F2D—C6D—C7D	110.3 (2)
F2B—C6B—C7B	110.4 (2)	F3D—C6D—C7D	113.8 (2)
O1B—C7B—O2B	128.8 (3)	O1D—C7D—O2D	128.9 (3)
O1B—C7B—C6B	116.1 (2)	O1D—C7D—C6D	114.7 (3)
O2B—C7B—C6B	115.0 (2)	O2D—C7D—C6D	116.3 (2)
			
C5A—N1A—C1A—C2A	0.6 (4)	C5C—N1C—C1C—C2C	1.1 (4)
N1A—C1A—C2A—C3A	−0.9 (4)	N1C—C1C—C2C—C3C	−0.3 (4)
C1A—C2A—C3A—C4A	0.5 (4)	C1C—C2C—C3C—C4C	−0.6 (5)
C2A—C3A—C4A—C5A	0.2 (4)	C2C—C3C—C4C—C5C	0.9 (4)
C1A—N1A—C5A—N2A	−178.4 (3)	C1C—N1C—C5C—N2C	179.2 (3)
C1A—N1A—C5A—C4A	0.0 (4)	C1C—N1C—C5C—C4C	−0.8 (4)
C3A—C4A—C5A—N2A	177.9 (3)	C3C—C4C—C5C—N2C	179.8 (3)
C3A—C4A—C5A—N1A	−0.5 (4)	C3C—C4C—C5C—N1C	−0.2 (4)
F2A—C6A—C7A—O2A	85.4 (3)	F3C—C6C—C7C—O1C	−154.3 (2)
F3A—C6A—C7A—O2A	−34.1 (3)	F1C—C6C—C7C—O1C	−32.5 (3)
F1A—C6A—C7A—O2A	−155.3 (2)	F2C—C6C—C7C—O1C	86.7 (3)
F2A—C6A—C7A—O1A	−92.0 (3)	F3C—C6C—C7C—O2C	28.9 (3)
F3A—C6A—C7A—O1A	148.5 (2)	F1C—C6C—C7C—O2C	150.7 (2)
F1A—C6A—C7A—O1A	27.4 (3)	F2C—C6C—C7C—O2C	−90.2 (3)
C5B—N1B—C1B—C2B	−1.3 (4)	C5D—N1D—C1D—C2D	0.5 (4)
N1B—C1B—C2B—C3B	0.5 (5)	N1D—C1D—C2D—C3D	−0.7 (4)
C1B—C2B—C3B—C4B	0.7 (5)	C1D—C2D—C3D—C4D	0.3 (5)
C2B—C3B—C4B—C5B	−1.3 (5)	C2D—C3D—C4D—C5D	0.2 (4)
C1B—N1B—C5B—N2B	−179.4 (3)	C1D—N1D—C5D—N2D	178.9 (3)
C1B—N1B—C5B—C4B	0.7 (4)	C1D—N1D—C5D—C4D	0.0 (4)
C3B—C4B—C5B—N2B	−179.3 (3)	C3D—C4D—C5D—N2D	−179.1 (3)
C3B—C4B—C5B—N1B	0.5 (4)	C3D—C4D—C5D—N1D	−0.4 (4)
F1B—C6B—C7B—O1B	31.0 (3)	F1D—C6D—C7D—O1D	37.8 (4)
F3B—C6B—C7B—O1B	152.5 (2)	F2D—C6D—C7D—O1D	−81.4 (3)
F2B—C6B—C7B—O1B	−88.5 (3)	F3D—C6D—C7D—O1D	159.6 (3)
F1B—C6B—C7B—O2B	−151.5 (2)	F1D—C6D—C7D—O2D	−144.0 (3)
F3B—C6B—C7B—O2B	−30.0 (3)	F2D—C6D—C7D—O2D	96.8 (3)
F2B—C6B—C7B—O2B	89.0 (3)	F3D—C6D—C7D—O2D	−22.2 (4)

### Hydrogen-bond geometry (Å, °)

**Table:**

*D*—H···*A*	*D*—H	H···*A*	*D*···*A*	*D*—H···*A*
N1A—H1NA···O2D^i^	0.92 (3)	1.91 (3)	2.809 (3)	168 (2)
N2A—H2NA···O1D^i^	1.02 (4)	1.79 (4)	2.795 (4)	169 (4)
N2A—H3NA···O1A^ii^	0.84 (3)	2.10 (3)	2.899 (3)	160 (3)
N1B—H1NB···O2C^iii^	0.97 (3)	1.75 (3)	2.704 (3)	166 (3)
N2B—H2NB···O1C^iii^	0.91 (4)	2.01 (4)	2.892 (4)	164 (3)
N2B—H3NB···O2B^iii^	0.86 (3)	2.07 (3)	2.858 (3)	154 (3)
N1C—H1NC···O1A^iv^	0.86 (3)	1.94 (3)	2.789 (3)	172 (3)
N2C—H2NC···O2A^iv^	0.90 (3)	1.93 (3)	2.827 (4)	177 (3)
N2C—H3NC···O2D^v^	0.94 (3)	2.04 (3)	2.894 (3)	150 (3)
N1D—H1ND···O2B^vi^	0.92 (3)	1.80 (3)	2.701 (3)	164 (2)
N2D—H2ND···O1B^vi^	0.95 (3)	1.97 (3)	2.878 (4)	160 (2)
N2D—H3ND···O2C^vii^	0.84 (3)	2.21 (3)	2.908 (3)	141 (3)
C1A—H1A···O2A	1.00 (3)	2.20 (3)	3.141 (3)	155 (2)
C1B—H1B···F3C^viii^	1.06 (3)	2.43 (3)	3.288 (4)	137 (2)
C1C—H1C···O1D^ix^	0.95 (2)	2.21 (2)	3.107 (3)	158 (2)
C4A—H4A···F1A^ii^	1.06 (3)	2.34 (3)	3.352 (3)	159 (2)
C4B—H4B···O1C	1.05 (3)	2.25 (3)	3.294 (4)	171 (2)
C4C—H4C···F3D^v^	0.93 (3)	2.50 (3)	3.365 (3)	155 (3)
C4D—H4D···O1B^ii^	1.08 (2)	2.18 (2)	3.204 (4)	159 (2)

Symmetry codes: (i) −*x*+1, −*y*+2, −*z*; (ii) *x*+1, *y*, *z*; (iii) −*x*+1, *y*−1/2, −*z*+1/2; (iv) −*x*+1, −*y*+1, −*z*; (v) *x*, *y*−1, *z*; (vi) −*x*+1, *y*+1/2, −*z*+1/2; (vii) −*x*+2, *y*+1/2, −*z*+1/2; (viii) *x*−1, *y*, *z*; (ix) *x*+1, *y*−1, *z*.
